# Effects of physical activity combined with different visual target presentation durations of ciliary-muscle training on visual acuity in children

**DOI:** 10.3389/fpubh.2023.1191112

**Published:** 2023-07-19

**Authors:** Sheng Zhou, Meng Zhang, Wenbin Zheng, Rongbin Yin, Gang Chen

**Affiliations:** ^1^School of Physical Education, Soochow University, Suzhou, China; ^2^Experimental Primary School of Suzhou Science and Technology Town, Suzhou, China

**Keywords:** physical activity, ciliary-muscle training, children, kinetic visual acuity, uncorrected distance visual acuity

## Abstract

**Purpose:**

This study aimed to identify the effect of different durations of visual target presentation during ciliary-muscle training on children's kinetic visual acuity (KVA), uncorrected distance visual acuity (UDVA), axial length, and accommodative facility.

**Methods:**

Based on the ciliary-muscle regulation mechanism, an intervention program involving ciliary-muscle training with different durations of visual target presentation combined with physical education classes was designed. The intervention aimed to determine the effect of different ciliary-muscle training durations on children's visual acuity. A total of 153 children aged 10–11 years from a school in Suzhou (a major city located in southeastern Jiangsu Province, East China) were enrolled as participants in this 32-week intervention study. This study measured the participants' UDVA and KVA before, during (after the 16th week), and after (after the 32nd week) the experimental intervention. The accommodative facility was measured during and after the intervention.

**Results:**

After 32 weeks of the intervention, the KVA and UDVA of each experimental group were significantly improved (*p* < 0.05). A high percentage in the improvement of KVA was observed in the 3-s and 1-s groups (25.53%, 21.74%), and the highest percentage in the improvement of UDVA was observed in the 3-s group (2.96%). Axial length increased significantly in all groups (*p* < 0.05), and there was a low percentage increase in the 1-s and 3-s groups (0.82%). The accommodative facility was significantly improved in all experimental groups, with a higher improvement percentage in the 3-s and 1-s groups (3.01% and 2.61%, respectively). After dividing the children in each group according to their visual acuity, the KVA of children in the 1-s group was significantly improved, the UDVA of children with myopia was significantly increased, and the accommodative facility of children with mild and moderate myopia was significantly improved. Moreover, the KVA, UDVA, and accommodative facility of children with mild and moderate myopia in the 3-s group were significantly improved. The KVA of children with emmetropia and the accommodative facility of children with mild and moderate myopia in the 5-s group were also significantly increased.

**Conclusion:**

In school physical education classes, the 1-s, 3-s, and 5-s ciliary-muscle regulating exercise could effectively improve the kinetic visual acuity, uncorrected distance visual acuity, and accommodative facility of children aged 10–11 years. Among them, the effects of the 1-s and 3-s durations are better than that of the 5-s duration, as it can reduce the growth rate of axial length and achieve better effects among children with mild and moderate myopia.

## 1. Introduction

Refractive errors are one of the most common causes of visual impairment. According to the World Report on Vision, there are currently ~2.6 billion people with myopia worldwide, including 312 million people under the age of 19 years (1). The eye health of children has always attracted increased attention, and myopia has become a major public health problem seriously affecting children's eye health. Myopia prevention and control have become one of the priorities in China. Several studies have indicated that physical activity is a protective factor for childrens' and adolescents' vision (2) and can reduce the risk of myopia or slow down its progression to some extent. Physical activity can improve and restore ciliary-muscle regulation and even induce short-term changes in ocular biometric parameters (3). However, different physical activity interventions have different effects on the prevention and control of myopia. There are differences in visual tasks in various physical activities, and the frequency and depth of ciliary-muscle adjustment to external stimuli are also different (4). For example, large-ball games were more effective than small-ball games in improving visual acuity (5). According to the characteristics of ball sports, a study hypothesized that an improvement in ocular biometric parameters may be related to the length of time required for each ciliary-muscle adjustment (6). The duration of a single visual target presentation during physical activity may be the reason for the difference in intervention effectiveness. At present, it is unclear whether the visual target presentation durations during physical activity affect the final outcome of the intervention.

It has been noted that there is a time course for any visual behavior, that each visual task requires a specific duration, and that the time required for humans to perform different types of visual behavior varies (7). Therefore, the duration of each presentation of a visual object is a key variable to control when designing physical activity interventions for myopia prevention and control. Although numerous studies have identified the importance of visual task duration, the optimal temporal range of visual tasks is still to be clarified. In particular, the control of the duration of a single visual task presentation in intervention programs is weak. In addition, people with myopia exhibit a lower rate of accommodation and longer time intervals on measures of accommodation facility compared to people with healthy vision (8). There may be differences in regulation function among visual acuity levels, as well as differences in the time required to recognize visual objects.

Therefore, this study explored and attempted to clarify the following questions through a 32-week intervention experiment: (1) What is the effect of visual tasks with different presentation durations during physical activity on children's visual acuity levels? The hypothesis is that visual tasks with different presentation durations during physical activity have a significant positive effect on children's visual acuity levels; (2) Whether the difference of visual task presentation durations in physical activity will lead to differences in the final intervention effect of prevention and control of myopia? For exactly how many seconds the single visual task presents would the intervention program work best? The hypothesis is that different presentation durations of visual task have different effects on children's visual acuity level. Furthermore, the single visual task presentation durations of 1 second to 3 second in a physical activity intervention for myopia prevention and control is more effective in improving the visual acuity of children; (3) What is the effect of different visual task presentation durations on the visual acuity of children with emmetropia, mild-to-moderate myopia and severe myopia, and do different visual task durations apply to children with different levels of vision? The hypothesis is that single visual task presentation durations of 1 s or 3 s in a physical activity intervention for myopia prevention and control are more effective in improving the visual acuity level of children with mild-to-moderate myopia.

## 2. Materials and methods

### 2.1. Participants and design

Before the experiment began, sample size estimation was conducted using G^*^Power. The sample size was determined based on an effect size of 0.25, statistical efficacy of 0.8, type I error probability (α) of 0.05, and the utilization of mixed methods ANOVA as the statistical analysis method. The calculation yielded a minimum required sample size of 76 students. Considering the difficulty of conducting different experimental interventions in the same class, in March 2021, four classes of 153 children aged 10–11 years were selected from Suzhou Science and Technology City Experimental Primary School using randomized whole-group unduplicated sampling. The children were classified by class to ensure no overlap between groups and randomly divided into 1-s group (*n* = 39), 3-s group (*n* = 38), 5-s group (*n* = 38), and control group (*n* = 38). One-way ANOVA was used to evaluate chi-square and homogeneity, which revealed that the differences in visual acuity-related indicators between the groups were not statistically significant (*p* > 0.05), consistent with the chi-square results obtained before the experiment, as shown in Table 1.

**Table 1:** Participants demographic characteristics (*N* = 153, Mean ± SD).

**Group (*N*)**	**KVA**	**UDVA**	**Axial length**	**Accommodative facility**
1 s (39)	0.46 ± 0.20	4.76 ± 0.29	24.29 ± 0.97	8.42 ± 2.68
3 s (38)	0.47 ± 0.26	4.73 ± 0.26	24.27 ± 1.09	8.65 ± 1.62
5 s (38)	0.48 ± 0.25	4.76 ± 0.31	24.24 ± 0.95	8.54 ± 2.24
Control group (38)	0.46 ± 0.24	4.77 ± 0.31	24.32 ± 0.92	8.43 ± 2.19
*F*-value	0.064	0.124	0.042	0.084
*P*-value	0.305	0.210	0.762	0.061

The inclusion criteria of eligible subjects in this study are as follows: (1) being healthy, with no physical diseases that are not compatible with physical activities; (2) uncorrected distance visual acuity (UDVA) ≥ 4.0, no wearing orthokeratology lenses, no astigmatism, amblyopia, hyperopia, and other eye pathological symptoms; and (3) no cognitive and motor dysfunction, and being able to successfully complete the experimental tasks according to language instructions.

The exclusion criteria for this research are as follows: (1) subjects with systemic, cardiopulmonary, and central nervous system diseases, or any musculoskeletal pathology accompanied by symptoms during the experiment; (2) UDVA < 4.0 in both eyes or with pathological eye diseases; (3) subjects whose kinetic visual acuity (KVA) could not be measured; and (4) subjects who could not continue to participate in the experiment due to school transfer during the experiment or who failed to meet the requirements of the experiment.

All measurements and experiments were carried out in the school. All participants and parents were informed about the content and purpose of the study and provided their signed informed consent. All procedures complied with the ethical standards of the *Declaration of Helsinki*, legal requirements, and international norms, and this study was approved by the Ethics Committee of Soochow University (No. SUA20201010H01).

### 2.2. Procedure and experimental manipulations

The study lasted for a total of 32 weeks and used a mixed experimental design of 4 (sight marker presentation 1 s, 3 s, 5 s, and no sight marker set) × 3 (time: pre-intervention, during, and post-intervention). The group was a between-subjects factor, and the test time point was a within-subjects factor. The experiment ensured that students in each class could participate in the same dose of physical activity during PE lessons. Subjects were tested for UDVA, KVA, axial length, and accommodative facility before, during (after 16 weeks of training), and after (after 32 weeks of training) the experimental intervention. The implementation of the physical education curriculum is carried out based on the national curriculum standards for Physical Education and Health.

#### 2.2.1. Viewpoint settings and types of physical activity

In this study, Ciliary-muscle training refers to the additional visual-target tracking and recognition tasks added to physical activities, so that the ciliary muscle can be exercised by seeing visual target information clearly under the designed guidance of seeing near to far or from far to near in physical activities. According to the classification theory of sports skills, this study chose closed sports skills including running, jumping, and throwing, and open skills involving football, basketball, and volleyball. Based on the purpose of the study and the characteristics of various skills and learning requirements, appropriate visual targets were added to these physical activities, which were designed in the form of hand-held cards, sports equipment (such as football, basketball, and sign barrel), or letters, numbers, and patterns that can be glued on the equipment. The presentation time of visual targets in each experimental group was 1 s, 3 s, and 5 s, respectively. The size of the visual target was designed according to the size of “distant vision 4.0” (9) and “near vision 4.0” (10) in the standard eye chart. The width of each side of the distant vision target was 43.64 mm and the stroke width was 8.73 mm, and the width of each side of the near vision target was 4.36 mm and the stroke width was 0.87 mm.

#### 2.2.2. Specific interventions

The experimental groups were set up as a 1-s group, 3-s group, and 5-s group. To strictly control the presentation duration of each visual target in ciliary-muscle training, the timekeeper of each group mainly used a stopwatch for timing, supplemented by the four-digit (e.g., 1,001 and 1,002) timing method of cardiopulmonary resuscitation. For each ciliary-muscle exercise, participants had to both see far and see near once; 1 ciliary-muscle exercise consists of 1 time for distant vision (for 1 s, 3 s, or 5 s) and 1 time for near vision (for 1 s, 3 s, or 5 s). Participants needed to complete 30 times for each intervention (11). Take running and basketball dribbling exercises as an example: (1) Running exercise: when the subjects started from the starting line and ran to the sign barrel, they first observed the near visual target pasted on the sign barrel. After observing for 1 s, 3 s, or 5 s, they made prescribed movements (such as high leg lifting, jumping, and back kicking) *in situ* according to the teacher's instruction. In this process, they were asked to raise their heads and look ahead to identify the distant visual target in 1 s, 3 s, or 5 s in the hands of the students in front of them. Then, they ran around the sign barrel and back to the starting point; (2) Basketball dribbling exercise: participants started from the starting line, dribbled the ball to the sign barrel, and then dribbled the ball with their left and right hands *in situ*. First, they observed the near visual target pasted on the sign barrel for 1 s, 3 s, or 5 s, looked far to identify the visual target on the distant visual frame for 1 s, 3 s, or 5 s, and then continued to dribble around the sign barrel. The intervention experiment lasted for 32 weeks, three times per week, and each intervention lasted for 40 min (Table 2).

**Table 2:** Experimental content.

**Classification**	**Projects**	**Exercise content**	**Visual target and equipment**	**Ciliary-muscle training**	**Experimental design and implementation considerations**
Closed motor skills	Run	Obstacle run, speed run, relay run, etc.	Sandbag, ball, baseball, solid ball, basketball, football, volleyball, bucket, frame, scaling ladder, digital card, letter card, text card, etc.	Emphasis of this training placed on the visual changing process from “visible” to “see details of the visual target clearly,” and from near to far or from far to near when participants were guided to track visual targets in running, jumping, and casting, which are expected to fully mobilize the ciliary muscle	(1) To ensure individual's safety; (2) regarding KVT, the change of distance in the exercise was the main concern; (3) ciliary-muscle training was flexibly designed and added according to the actual teaching content of each physical education class; (4) according to the learning characteristic of children aged between 10 and 11 years, progressing step by step, from easy to difficult and from simple to complex.
	Jump	Height adjustment, long jump, etc.			
	Cast	Sandbag, softball, solid ball			
Open motor skills	Basketball	Dribbling the basketball, shooting *in situ*, passing and catching the ball			
	Soccer	Kicking the ball with the inside foot, catching the ball, dribbling the ball in front of the instep			
	Volleyball	Moving and dribbling the ball with the hands, underhand serving, etc.			

### 2.3. Outcome measures

This study measured the participants' UDVA and KVA before the experimental intervention, during the experimental intervention (after the 16th week), and after the experimental intervention (after the 32nd week). The accommodative facility was measured during and after the intervention. To minimize error, the detection methods and process were strictly carried out in accordance with the standards, and the same person conducted all the measurements and recording of various data before and after the experiment.

#### 2.3.1. Uncorrected distance visual acuity

Participants' UDVA was completed by the full-time school doctor using the lightbox of the current “Standard Logarithmic Visual Acuity Chart (GB111533-2011)” for testing. Each subject was guided to stand at the marking line, and one side of the glasses was gently covered with a black blindfold without oppressing the eyeball, following the principle of the first right eye and then the left eye. Children who wore glasses or contact lenses took off their glasses and rested for a while before performing the UDVA test. The inspector checked the visual acuity chart line-by-line from the top to the bottom with a baton, and participants were required to immediately speak out or point out the direction indicated by the visual target. The value displayed in the line where the child's correct answer was located was taken as the final value of their UDVA, and the minimum measurement result of their left and right eye was taken as the final UDVA value.

#### 2.3.2. Kinetic visual acuity

KVA was tested with XP.14-TD-J905, the KVA detector produced by Shanghai Hump Automation Technology Co., Ltd. that conforms to the national standard GB18463-2001 of the People's Republic of China. The KVA range was between 0.1 and 1.6, and the higher the value, the better the KVA. Before the eyesight test, the tester explained the operation method, and all subjects were given instructions and a demonstration of the operation steps of the detector twice to learn and master it. At the beginning of the formal test, each child was tested three times in succession, with an interval of 30 s between each test. The participants sat in front of the detector and placed their eyes close to the window of the measuring eyepiece. After the tester swiped the card, the instrument started the testing state, and a Landolt ring was displayed in the eyepiece of the instrument with a different opening direction each time (mainly four directions: up, down, left, and right). This simulated that the ring moved from 50 m away from the participant at a speed of 30 km/h, from far to near. The students were instructed to rapidly press the joystick to show the orientation that they identified on the Landolt ring. When the subject operated correctly, the LCD screen on the instrument automatically displayed the KVA value. The tester noted the value in the special record form and swiped the card to do the next test. The average of the three recorded values was the subject's final KVA value.

#### 2.3.3. Axial length

Participants' axial length was measured using IOL Master 500 (Zeiss). The participants were asked to put their lower jaw on the jaw support of the device, put their forehead close to the forehead support, and adjust their head position to maintain the eye to be examined on the designated target. Then, the instrument automatically measured the length of the subject's axial length five consecutive times, and the averaged results were recorded.

#### 2.3.4. Binocular accommodative facility

Binocular Accommodative Facility was measured using ± 2.00 D Flipper lens. Before the start of the test, the PE teacher in charge of the class organized for the test subject to enter the test site, ensuring that there was sufficient light and no distracting factors. The subject sat in front of the desk with the visual target reading card 40 cm away from the eyes on the desk. Then, the ± 2.0 D Flipper lens was alternately placed in front of the eyes to identify the visual target through the + 2.0 D lens. After clear identification, this was quickly switched to the −2.0 D lens to identify the visual target again. A test cycle included shifts from a positive lens to a negative lens and back to a positive lens. The number of cycles in which the subject could see visual targets clearly within 1 min was recorded in the form of cycles/min. The higher the number of cycles of the accommodative facility, the better the adjustment ability.

### 2.4. Statistical analysis

Statistical analysis was conducted using SPSS 26.00. The data of UDVA, KVA, axial length, and accommodative followed a normal distribution and were expressed as mean ± standard deviation (M ± SD). A repeated measures ANOVA was used to conduct inferential analyses of the group (visual scale presentation for 1 s, 3 s, 5 s, and no visual scale setting) and time (time: before, during, and after the intervention) effects of the intervention to test for group and time effects, and time and group interaction effects. ηp^2^ was used to evaluate the significance of the main effect and interaction, in which ηp^2^ < 0.06 was a small effect, 0.06 ≤ ηp^2^ ≤ 0.14 was a medium effect, and ηp^2^ > 0.14 was a large effect. The significance level is α = 0.05. A paired-sample *t*-test was used to compare the changes in the mean values of each visual acuity index before, during, and after the experiment. Children were grouped by UDVA level to determine the changes in KVA, axial length, and accommodative facility at different visual acuity levels by the three visual scale presentation time values.

## 3. Results

### 3.1. Intervention effect analysis of different ciliary-muscle training durations on KVA

Repeated measure analysis of variance showed that the main effect of time was significant (*F* = 9.261, *p* < 0.05, ηp^2^ = 0.111), and the interaction of time × group was significant (*F* = 5.986, *p* < 0.05, ηp^2^ = 0.108). Further simple effect analysis of time × group interaction showed that there was no significant difference in KVA between the experimental groups and the control group in the pre-test and mid-test stage (F_T1_ = 0.064, F_T2_ = 1.301, *p* > 0.05), but the value of KVA of the experimental groups was significantly higher than that of the control group in the post-test stage (F_T3_ = 5.775, *p* < 0.05). These results indicate that different visual task durations can affect the KVA at the time point after the experiment (Table 3).

**Table 3:** Changes of KVA.

**Group (*N*)**	**Pre-test (T1)**	**Mid-test (T2)**	**Post-test (T3)**	***F-*value**	***P*-value**	**ηp^2^**
1 s (39)	0.46 ± 0.20	0.50 ± 0.21a	0.56 ± 0.22ab	7.658	< 0.05	0.094
3 s (38)	0.47 ± 0.26	0.52 ± 0.25a	0.59 ± 0.26ab	10.491	< 0.05	0.124
5 s (38)	0.48 ± 0.25	0.51 ± 0.24	0.57 ± 0.30a	5.309	< 0.05	0.067
Control group (38)	0.46 ± 0.24	0.43 ± 0.22	0.38 ± 0.19a	4.944	< 0.05	0.063
*F*-value	0.064	1.301	5.775			
*P*-value	>0.05	>0.05	< 0.05			
ηp^2^	0.001	0.026	0.104			

*p* < 0.05 when “a” was compared with T_1_; *p* < 0.05 when “b” was compared with T_2_.

The paired-sample *t*-test was carried out to further observe the changes in the participants' KVA in each group. It was found that the post-test results of the 1-s, 3-s, and 5-s groups were significantly improved compared with that of the pre-test (*p* < 0.05). The post-test results of the 1-s group and 3-s group were significantly improved compared with the mid-test (*p* < 0.05), but there was no significant difference between the post-test and the mid-test of the 5-s group (*p* > 0.05).

**Figure 1:**
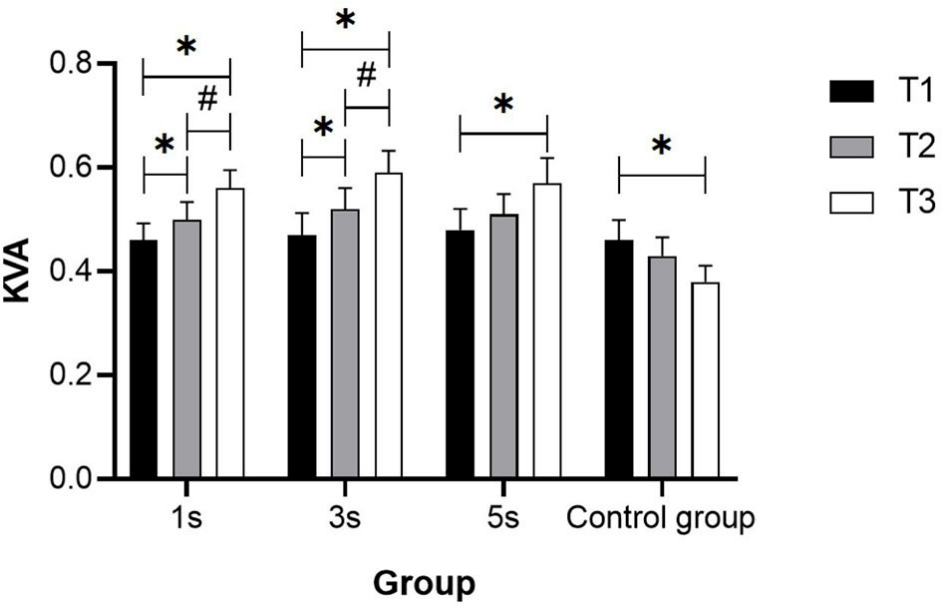
KVA change trend in groups with different ciliary-muscle training durations. ^*^Represents significant difference compared with T1 (*p* < 0.05); ^#^Represents significant difference compared with T2 (*p* < 0.05).

In the 1-s and 3-s groups, the data of the mid-test were significantly higher than that of the pre-test (*p* < 0.05), but there was no significant difference between the mid-test and the pre-test in the 5-s group (*p* > 0.05). In the control group, there was no significant difference between the mid-test and the pre-test, and among the post-test and mid-test (*p* > 0.05), there was a significant decrease in the post-test compared with the pre-test (*p* < 0.05) (Figure 1).

The percentage improvement of KVA in each experimental group was calculated according to the formula [(mean T3-mean T1)/mean T1 × 100%], and the result was 21.74% in the 1-s group, 25.53% in the 3-s group, and 18.75% in the 5-s group, respectively.

### 3.2. Intervention effect analysis of different ciliary-muscle training durations on UDVA

Repeated measure analysis of variance showed that the main effect of time was significant (*F* = 10.109, *p* < 0.05, ηp^2^ = 0.120) and the interaction of time × group was significant (*F* = 6.451, *p* < 0.05, ηp^2^ = 0.116). Further simple effect analysis of time × group interaction showed that there was no significant difference in UDVA between the experimental groups and the control group in the pre-test and mid-test stage (F_T1_ = 0.124, F_T2_ = 0.636, *p* > 0.05), but the value of UDVA of the experimental groups was significantly higher than that of the control group in the post-test stage (*F* = 3.695, *p* < 0.05) (Table 4).

**Table 4:** Changes of UDVA.

**Group (*N*)**	**Pre-test (T1)**	**Mid-test (T2)**	**Post-test (T3)**	***F*-value**	***P*-value**	**ηp^2^**
1 s (39)	4.76 ± 0.29	4.81 ± 0.32a	4.87 ± 0.32ab	7.675	< 0.05	0.94
3 s (38)	4.73 ± 0.26	4.79 ± 0.27a	4.87 ± 0.30ab	11.262	< 0.05	0.132
5 s (38)	4.76 ± 0.31	4.80 ± 0.30	4.87 ± 0.31ab	6.383	< 0.05	0.79
Control group (38)	4.77 ± 0.31	4.72 ± 0.31a	4.68 ± 0.32a	5.485	0.05	0.69
*F*-value	0.124	0.636	3.695			
*P*-value	>0.05	>0.05	< 0.05			
ηp^2^	0.002	0.013	0.069			

*p* < 0.05 when “a” was compared with T_1_; *p* < 0.05 when “b” was compared with T_2_.

**Figure 2:**
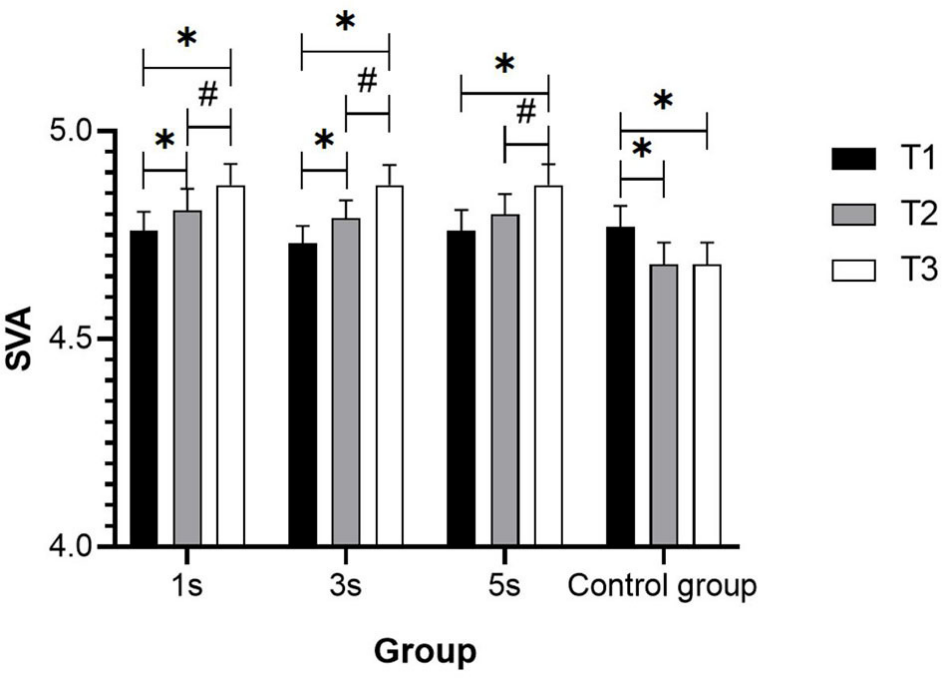
SVA change trend in groups with different ciliary-muscle training durations. *Represents significant difference compared with T1 (*p* < 0.05); ^#^Represents significant difference compared with T2 (*p* < 0.05).

It was found that the post-test results of the 1-s group and the 3-s group were significantly higher than that of their pre-test, these post-test results were higher than that of the mid-test, and the mid-test data were higher than that of the pre-test (*p* < 0.05). In the 5-s group, the post-test value was significantly higher than that of the pre-test and mid-test (*p* < 0.05), but there was no significant difference in results between the mid-test and the pre-test (*p* > 0.05). In the control group, there was a significant decrease in the mid-test results and the post-test results compared with that of the pre-test (*p* < 0.05), but there was no significant difference between the post-test and the mid-test (*p* > 0.05) (Figure 2).

The percentage improvement of UDVA of each experimental group was calculated according to the formula [(mean T3-mean T1)/mean T1 × 100%], and the result was 2.31% of the 1-s group, 2.96% in the 3-s group, and 2.31% in the 5-s group, respectively.

### 3.3. Intervention effect analysis of different ciliary-muscle training durations on axial length

Repeated measures analysis of variance showed that the main effect of time was significant (*F* = 121.516, *p* < 0.05, ηp^2^ = 0.622). Further simple effect analysis showed that there was no significant difference in axial length between the experimental groups and the control group in the pre-test, mid-test, and post-test (*F*_T1_= 0.042, *F*_T2_ = 0.046, *F*_T3_ = 0.073, *p* > 0.05). The paired-sample *t*-test showed that the post-test results in each group were significantly higher than that of the pre-test and the mid-test, and the data of the mid-test were higher than that of the pre-test in each group (*p* < 0.05) (Table 5).

**Table 5:** Change in axial length.

**Group (*N*)**	**T1**	**T2**	**T3**	***F*-value**	***P*-value**	**ηp^2^**
1 s (39)	24.29 ± 0.97	24.35 ± 0.97a	24.49 ± 0.97ab	28.885	< 0.05	0.281
3 s (38)	24.27 ± 1.09	24.35 ± 1.09a	24.47 ± 1.11ab	25.879	< 0.05	0.259
5 s (38)	24.24 ± 0.95	24.31 ± 0.95a	24.46 ± 0.94ab	31.950	< 0.05	0.302
Control group (38)	24.32 ± 0.92	24.40 ± 0.92a	24.56 ± 0.97ab	36.787	< 0.05	0.332
*F*-value	0.042	0.046	0.073			
*P*-value	> 0.05	> 0.05	> 0.05			
ηp^2^	0.001	0.001	0.001			

*p* < 0.05 when “a” was compared with T_1_; *p* < 0.05 when “b” was compared with T_2_.

The percentage growth of axial length in each experimental group was calculated according to the formula [(mean T3-mean T1)/mean T1 × 100%], and the result was 0.82% in the 1-s group, 0.82% in the 3-s group, 0.91% in the 5-s group, and 0.99% in the control group, respectively.

### 3.4. Intervention effect analysis of different ciliary-muscle training durations on accommodative facility

Repeated measures analysis of variance showed that the main effect of time was significant (*F* = 8.676, *p* < 0.05, ηp^2^ = 0.055), and the interaction of time × group was significant (*F* = 6.113, *p* < 0.05, ηp^2^ = 0.110). Further simple effect analysis of the time × group interaction showed that there was no significant difference in the accommodative facility between the experimental groups and the control group in T_2_ and T_3_ (F_T2_ = 0.084, F_T3_ = 0.523, *p* > 0.05). This indicates that the different visual task durations have no influence on the accommodative facility at each time point. The paired-sample *t*-test showed that the results of the post-test of each experimental group were significantly higher than that of the mid-test (*p* < 0.05), and the post-test data of the control group were significantly lower than that of the mid-test (*p* < 0.05) (Table 6).

**Table 6:** Change of children's accommodative facility.

**Group (*N*)**	**T2**	**T3**	***F*-value**	***P-*value**	**ηp^2^**
1 s (39)	8.42 ± 2.68	8.64 ± 2.58b	7.677	< 0.05	0.049
3 s (38)	8.65 ± 1.62	8.91 ± 1.95b	10.905	< 0.05	0.068
5 s (38)	8.54 ± 2.24	8.70 ± 2.26b	3.926	< 0.05	0.026
Control group (38)	8.43 ± 2.19	8.26 ± 2.32b	4.607	< 0.05	0.030
*F*-value	0.084	0.523			
*P*-value	>0.05	>0.05			
ηp^2^	0.002	0.010			

*p* < 0.05 when “b” was compared with T_2_.

The percentage improvement of the accommodative facility in each experimental group was calculated according to the formula [(mean T3-mean T1)/mean T1 × 100%], and the result was 2.61% in the 1-s group, 3.01% in the 3-s group, and 1.87% in the 5-s group, respectively.

### 3.5. Intervention effect analysis of children with different visual acuity

#### 3.5.1. Intervention effect on children's visual acuity in the 1-s group

According to the *Technical Guidelines for Childrens' and Adolescents' Myopia Prevention and Control*, children and adolescents over 6 years of age with UDVA < 5.0 are myopic. Among them, a UDVA of 4.9 was considered mild myopia, from 4.6 to 4.8 as mild or moderate myopia, and lower than 4.5 as severe myopia. Owing to the small sample size in this study, the UDVA ≥ 5.0 was considered as emmetropia, 4.6–4.9 as mild or moderate myopia, and ≤ 4.5 as severe myopia, based on which further analysis was conducted.

The KVA, UDVA, axial length, and accommodative facility of children with emmetropia, mild or moderate, and severe myopia were measured in the 1-s group, and the data were analyzed by repeated measures analysis of variance, which showed that the main effects of time were significant (*p* < 0.05). The paired-sample *t*-test on children's KVA showed that the results of the post-test among children with emmetropia were significantly improved compared with that of the pre-test and mid-test (*p* < 0.05), but there was no significant difference between the mid-test and the pre-test (*p* > 0.05). Among children with severe myopia, the post-test data were significantly higher than that of the pre-test, and also significantly higher than that of the mid-test (*p* < 0.05). The UDVA of children with mild, moderate, and severe myopia was significantly improved in the post-test compared with the pre-test (*p* < 0.05), and that of children with severe myopia was significantly improved in the post-test compared with the pre-test and mid-test (*p* < 0.05). The axial length of children with different visual acuity was significantly improved in the post-test compared with the pre-test and mid-test, and in the mid-test compared with the pre-test (*p* < 0.05). Moreover, the accommodative facility of children with mild and moderate myopia increased significantly compared with that of the mid-test (*p* < 0.05) (Table 7).

**Table 7:** Change of children's visual acuity in the 1-s group.

**Visual indicator**	**Visual acuity**	**Pre-test (T1)**	**Mid-test (T2)**	**Post-test (T3)**	***F/T*-value**	***P*-value**
KVA	Emmetropia (10)	0.55 ± 0.19	0.60 ± 0.23	0.68 ± 0.26ab	6.340	< 0.05
	Mild and moderate myopia (19)	0.47 ± 0.15	0.50 ± 0.15	0.55 ± 0.16a	3.700	< 0.05
	Severe myopia (10)	0.34 ± 0.26	0.40 ± 0.26	0.47 ± 0.26ab	6.018	< 0.05
UDVA	Emmetropia (10)	5.11 ± 0.07	5.15 ± 0.11	5.19 ± 0.11	1.373	>0.05
	Mild and moderate myopia (19)	4.78 ± 0.11	4.84 ± 0.19	4.91 ± 0.24a	6.503	< 0.05
	Severe myopia (10)	4.36 ± 0.08	4.39 ± 0.13	4.49 ± 0.13a	3.602	< 0.05
Axial length	Emmetropia (10)	23.65 ± 0.72	23.72 ± 0.74a	23.86 ± 0.76ab	11.548	< 0.05
	Mild and moderate myopia (19)	24.42 ± 0.98	24.48 ± 0.98a	24.61 ± 0.99ab	17.033	< 0.05
	Severe myopia (10)	24.67 ± 0.96	24.76 ± 0.93a	24.91 ± 0.87ab	14.331	< 0.05
Accommodative facility	Emmetropia (10)		10.45 ± 2.78	10.55 ± 2.58	0.541	>0.05
	Mild and moderate myopia (19)		7.90 ± 1.88	8.18 ± 1.83b	8.609	< 0.05
	Severe myopia (10)		7.40 ± 3.03	7.60 ± 3.02	2.163	>0.05

*p* < 0.05 when “a” was compared with T_1_; *p* < 0.05 when “b” was compared with T_2_.

#### 3.5.2. Intervention effect on children's visual acuity in the 3-s group

The KVA, UDVA, axial length, and accommodative facility of children with emmetropia, mild or moderate, and severe myopia were measured in the 3-s group, and repeated measures analysis of variance showed that the main effects of time were significant (*p* < 0.05). The paired-sample *t*-test showed that children's KVA in the post-test was significantly improved among children with emmetropia compared with the pre-test (*p* < 0.05). In children with mild and moderate myopia, the post-test data were significantly higher than that of the pre-test and also significantly higher than that of the mid-test (*p* < 0.05). Moreover, the UDVA of children with emmetropia was significantly higher in the post-test than that in the pre-test and the mid-test (*p* < 0.05). Axial length in children with different visual acuity levels increased significantly in the post-test and mid-test compared with the pre-test (*p* < 0.05). Among children with mild, moderate, and severe myopia, axial length increased significantly in the post-test compared with the mid-test (*p* < 0.05). The accommodative facility of children with mild and moderate myopia increased significantly in the post-test compared with the mid-test (*p* < 0.05) (Table 8).

**Table 8:** Change of children's visual acuity in the 3-s group.

**Visual indicator**	**Visual acuity**	**Pre-test (T_1_)**	**Mid-test (T_2_)**	**Post-test (T_3_)**	***F/T*-value**	***P-*value**
KVA	Emmetropia (8)	0.58 ± 0.25	0.64 ± 0.23	0.73 ± 0.23a	3.212	0.05
	Mild and moderate myopia (22)	0.48 ± 0.26	0.54 ± 0.24a	0.63 ± 0.25ab	10.068	< 0.05
	Severe myopia (8)	0.34 ± 0.26	0.34 ± 0.23	0.34 ± 0.18	0.025	>0.05
UDVA	Emmetropia (8)	5.05 ± 0.05	5.03 ± 0.14	5.16 ± 0.13ab	3.411	< 0.05
	Mild and moderate myopia (22)	4.75 ± 0.12	4.85 ± 0.17a	4.92 ± 0.20a	14.074	< 0.05
	Severe myopia (8)	4.36 ± 0.15	4.40 ± 0.17	4.45 ± 0.19	1.245	>0.05
Axial length	Emmetropia (8)	23.58 ± 0.89	23.64 ± 0.89a	23.71 ± 0.94a	6.909	< 0.05
	Mild and moderate myopia (22)	24.33 ± 1.08	24.40 ± 1.07a	24.53 ± 1.08ab	28.620	< 0.05
	Severe myopia (8)	24.80 ± 1.06	24.91 ± 1.04a	25.05 ± 1.04ab	20.135	< 0.05
Accommodative facility	Emmetropia (8)		9.94 ± 1.84	10.13 ± 1.98	0.517	>0.05
	Mild and moderate myopia (22)		8.68 ± 1.32	9.02 ± 1.84b	4.701	< 0.05
	Severe myopia (8)		7.25 ± 1.07	7.38 ± 1.25	0.230	>0.05

*p* < 0.05 when “a” was compared with T_1_; *p* < 0.05 when “b” was compared with T_2_.

#### 3.5.3. Intervention effect on children's visual acuity in the 5-s group

The KVA, UDVA, axial length, and accommodative facility of children with emmetropia, mild or moderate, and severe myopia were measured in the 5-s group, and repeated measures analysis of variance showed that the main effects of time were significant (*P* < 0.05). The paired sample *t*-test showed that children's KVA in the post-test was significantly improved among children with emmetropia compared with the pre-test (*p* < 0.05). Moreover, the UDVA of children with emmetropia was significantly higher in the post-test than that in the pre-test and the mid-test (*p* < 0.05). Axial length in children with different visual acuity levels increased significantly in the post-test and mid-test compared with the pre-test (*p* < 0.05). Among children with mild and moderate myopia, axial length increased significantly in the post-test compared with the mid-test, and in the mid-test compared with the pre-test (*p* < 0.05). For children with severe myopia, axial length increased significantly in the post-test compared with the pre-test (*p* < 0.05). The axial length of children with emmetropia and severe myopia was significantly increased at all stages (*p* < 0.05), and that of children with mild and moderate myopia was significantly improved compared with the pre-test (*p* < 0.05). The accommodative facility of children with mild and moderate myopia increased significantly in the post-test compared with the mid-test (*p* < 0.05) (Table 9).

**Table 9:** Change of children's visual acuity in the 5-s group.

**Visual indicator**	**Visual acuity**	**Pre-test (T_1_)**	**Mid-test (T_2_)**	**Post-test (T_3_)**	***F/T*-value**	***P*-value**
KVA	Emmetropia (11)	0.71 ± 0.27	0.74 ± 0.23	0.84 ± 0.31a	4.018	< 0.05
	Mild and moderate myopia (15)	0.49 ± 0.13	0.52 ± 0.13	0.57 ± 0.20	2.562	>0.05
	Severe myopia (12)	0.26 ± 0.08	0.29 ± 0.12	0.33 ± 0.15	1.882	>0.05
UDVA	Emmetropia (11)	5.16 ± 0.05	5.09 ± 0.16	5.13 ± 0.13	1.225	>0.05
	Mild and moderate myopia (15)	4.75 ± 0.13	4.85 ± 0.16a	4.93 ± 0.21a	6.915	>0.05
	Severe myopia (12)	4.41 ± 0.09	4.47 ± 0.20	4.55 ± 0.26a	3.205	0.05
Axial length	Emmetropia (11)	23.46 ± 0.69	23.53 ± 0.69a	23.74 ± 0.71ab	11.248	< 0.05
	Mild and moderate myopia (15)	24.65 ± 0.97	24.71 ± 1.00	24.85 ± 1.02a	6.068	< 0.05
	Severe myopia (12)	24.47 ± 0.68	24.53 ± 0.67a	24.63 ± 0.67ab	3.195	0.05
Accommodative facility	Emmetropia (11)		9.41 ± 1.83	9.50 ± 1.87	0.0934	>0.05
	Mild and moderate myopia (15)		8.17 ± 2.30	8.40 ± 2.39b	8.395	< 0.05
	Severe myopia (12)		8.21 ± 2.47	8.33 ± 2.40	1.927	>0.05

*p* < 0.05 when “a” was compared with T_1_; *p* < 0.05 when “b” was compared with T_2_.

## 4. Discussion

### 4.1. Effects of visual tasks with different presentation durations during physical activity on children's visual acuity levels and ciliary-muscle accommodative ability

Approximately 90% of visual problems among school-age children are caused by myopia. The progression of myopia is accompanied by changes in a series of accommodative parameters of the ciliary muscle, such as the decreased and disordered accommodative function of the ciliary muscle (12), which is one of the major reasons for the development of myopia. The early stage of myopia mainly results from the continuous contraction and spasm of the ciliary muscle, which leads to temporary myopia because the lens cannot recover in time when people try to see something far away. If this situation cannot be corrected in time, it is easy to progress into true myopia. Restoring ciliary-muscle accommodative function is the fundamental means to prevent and control myopia. Therefore, in the early stage of myopia, it is particularly important to carry out ciliary-muscle training for the improvement of its regulating function (13). The results of this study showed that after 32 weeks of intervention, children's KVA and UDVA in each experimental group were significantly improved, which confirmed again that physical activities designed according to the ciliary-muscle accommodating principle are an effective intervention to prevent myopia and slow down the process of myopia (4, 14). KVA plays an important role in improving the UDVA of children and adolescents (15). KVA, the ability to perceive details of objects moving backward and forward toward the eye, relies greatly on the ciliary muscle and is closely linked to its regulation. Previous studies have shown that although the specific function of KVA and UDVA is different, they both depend on the ciliary-muscle activity. UDVA is the basis for KVA, and improved KVA promotes improved UDVA. UDVA is the basis of KVA, and the improvement of KVA can promote the improvement of UDVA. However, the process of quantitative change to qualitative change requires a certain time process for the internal mechanism of the refractive system to adapt to each other (16). In addition, physical activity with additional kinetic visual tasks also improved children's binocular accommodative facility, with a significant time-dominant effect, validating the study hypothesis further.

At the end of the 19th century, the concept of visual training was put forward in the fields of medicine and optometry. Visual training can improve children's visual function. Visual training is mainly based on the eye muscles and vision conditions of the targeted arrangement of exercises. There are many types of visual training, among which regulating function training is essential to stimulate the ciliary muscle to complete the contraction and relaxation alternately through the aid of lenses or visual targets (17). The goal is to achieve the effect of improving the regulation function of the ciliary muscle. In fact, eyes moving far and near following objects in physical activities are consistent with visual training. This study used alternating visual tracking of distance and near during physical activity to guide the human eye in active recognition, effectively mobilizing ciliary-muscle contraction and diastole, relieving muscle spasms, and enhancing regulatory flexibility. Compared with traditional therapeutic visual training, physical activity with additional ciliary-muscle regulating function training is more interesting. The sports selected for this study are commonly practiced in school and are of interest to children with a certain degree of athletic foundation and children are more willing to accept it. This ensured that negative emotions caused by the repetitive and boring tasks were avoided. In addition, the integration of physical activity and ciliary-muscle regulating training is much more convenient to carry out and implement, which is conducive to maximizing the effectiveness of myopia prevention and control and greatly increases childrens' and adolescents' opportunities for visual health promotion.

The results of this study provide ideas for designing visual training combining physical activity in the future. Some researchers believe that traditional sports training usually focused on improving individuals' limbs and trunk muscles, while visual training was conducted to improve the function of eye muscles. Although there are differences between visual training and sports training in form, the theoretical basis of the two sports training is the same, that is, both of them are derived from basic sports training theories, such as motion adaptability theory, and follow basic sports training principles, such as reasonable arrangement of exercise load (18). Therefore, physical activities designed to prevent and control children's myopia also need to specify a reasonable amount of exercise to quantify the intervention program. Based on previous studies, this study explored the optimal presentation duration of visual targets in ciliary-muscle training to improve the implementation of intervention programs.

The results of the study showed that the eye axis continued to grow over time in all groups of children during the current experimental period. It is now generally accepted that true myopia is irreversible, that the growth of the eye axis is essentially a deformation of the eye, and that the changes in the eye axis that accompany myopia are difficult to reverse. The abnormal accommodative function of the ciliary muscle causes the choroid to pull forward and backward, and then affects the shape of the sclera, resulting in changes in eye axial length (19, 20). It is known that axial length is an important index reflecting the growth and development of the eyeball and the change in visual acuity, which increases with age. The growth rate of the eye axis in children with myopia is faster than that of children with emmetropia (21). The failure to meet the experimental expectation of the axial change may also be because the subjects in this research were in the critical period of preparation for the entrance exam, and short-term ciliary-muscle training was difficult to offset the negative effects of the long-term screen and near work under the high pressure of schoolwork. Research has shown that relief of ciliary-muscle tension would change choroidal tension and improve the mechanical properties of the ciliary muscle, thereby reducing the growth rate of axial length (22). To achieve a benign change of the eye axis, more in-depth experimental research and long-term follow-up observation have to be conducted in the future.

### 4.2. Differences in the effects of visual tasks with different presentation durations in physical activity on the improvement of children's visual acuity levels

Further analysis revealed that the effect of the visual task on children's KVA and UDVA differed across presentation time values during the physical activity. There were no significant differences in the characteristics of the demographic variables and physical and mental health status of the children in this study, and only the presentation time values of the visual tasks during physical activity differed throughout the experiment, while the other variables were controlled or conditionally consistent. Therefore, the reason for the differences in the study results was the duration of the single visual task. The duration of the visual task is an important factor of a physical activity program for the prevention and control of myopia in children and ultimately determines the effect of the improvement in children's visual acuity levels.

Through the analysis of the results, it was found that children's KVA and UDVA had been significantly improved in the mid-experiment in the 1-s and 3-s groups, while there was a lag effect in the 5-s group, which suggested that the 1-s and 3-s training duration had a better effect on the improvement of children's visual acuity. Further calculation of the percentage improvement in each experimental group showed that the 3-s group had the best result. The accommodative facility of each experimental group was significantly improved, while that of the control group was significantly reduced, The 3-s group has the highest increase in percentage of accommodative facility, while the 5-s group has the lowest increase in percentage. In addition, during the experiment of this study, the eye axis of each group continued to grow, but the percentage of axial growth rate in the 1-s group and the 3-s group was lower than that of the 5-s group and the control group, indicating that the 1-s and 3-s ciliary-muscle exercise may slow down the growth rate of the eye axial length.

Therefore, a presentation duration of 3 s is most reasonable for the additional single visual task in physical activity, especially for children aged 10–11 years, and the results of this study are mainly attributed to the following reasons. The ciliary muscle is mainly innervated by dense cholinergic parasympathetic nerve endings, and there is also evidence to support that it may be subjected to sympathetic innervation. Parasympathetic innervation increases more rapidly when seeing something near, with rapid focusing in 1 or 2 s, while sympathetic innervation is slower for seeing something far away (23, 24). Both sympathetic and parasympathetic innervations require a certain period of time. The 3-s duration of visual target presentation and the depth and intensity of its stimulation on the ciliary muscle are moderate, with which the ciliary muscle can completely contract and relax, thus helping to enhance visual accommodative ability, the coordination of the convergence system of the human eye, and the interaction between sympathetic and parasympathetic nerves (13). The 5-s group had a longer interval of ciliary-muscle practice, although there was enough time to identify the visual target, the slow switching speed and the distraction of children's attention may lead to the weakening of the overall intervention effect of ciliary-muscle regulating training.

### 4.3. Effects of visual tasks with different presentation durations during physical activity on children with different levels of visual acuity

To further explore the effect of ciliary-muscle training combined with physical activity on children's visual acuity, this study examined changes in all participants' KVA, UDVA, axial length, and accommodative facility. The results indicated that with 1-s target presentation, children's KVA of all vision levels was significantly improved, the UDVA of children with myopia was remarkably improved, and the accommodative facility of children with mild and moderate myopia was significantly increased. The KVA, UDVA, and accommodative facility of children with mild and moderate myopia had the best improvement when their visual target was presented for 3 s. In addition, the KVA of children with emmetropia and the accommodative facility of children with mild and moderate myopia were significantly improved when their visual targets were presented for 5 s. Generally, The 1-s and 3-s duration of visual target presentation are more suitable for children with mild and moderate myopia, which can effectively relieve ciliary-muscle spasms and improve their KVA, UDVA, and accommodative facility.

This study investigated the differences in the effects of physical activity combined with ciliary-muscle training at different values of visual task presentation on visual acuity in 10–11-year-old children, providing new evidence to fully reveal the relationship between physical activity itself and myopia improvement. It suggests that physical activity, especially physical activity that reinforces ciliary-muscle training, is a proven way to improve visual acuity in children. Most importantly, this study identified the optimal range of visual task presentation times for physical activity in the prevention and control of myopia in children, providing a basis for the design of future visual training incorporating physical activity and is a reference for the prescription of physical activity myopia prevention and control programs. It also reveals that the design of future interventions for the prevention and control of myopia needs to pay more attention to the rational arrangement of motor loads so that intervention programs that improve the final implementation effect are used. In addition, the experiments in this study were implemented in real school scenarios, making the study highly ecologically valid and the findings more credible. The findings of this investigation are of great value to the practice of physical education activities in schools. Schools and teachers can make full use of the results and conclusions drawn from this study to set up visual exercises in school physical education classes in a scientific and reasonable manner to promote the healthy development of children's visual acuity.

Although great effort was made, the present study has some limitations. First, considering the safety concerns of some children's parents and the guarantee of no optometry interruption and restriction on children's daily life, this study did not use mydriasis and other paralytic optometry methods to obtain gold standard data, such as spherical equivalent power. Second, due to the negative impact of the global coronavirus disease 2019 (COVID-19), only 10–11-year-old children were selected for the experiment, but no comparative study of multi-population or larger samples was carried out. It is a long-term process to improve and control children's myopia through additional ciliary-muscle regulating exercise incorporated into physical activities, which requires long-term experimental monitoring. In future studies, children of various ages should be selected to explore the most desirable exercising combination of physical activity and ciliary-muscle regulation.

## 5. Conclusion

The present study showed that the 1-s, 3-s, and 5-s ciliary-muscle training exercises could effectively improve children's KVA, UDVA, and ciliary-muscle accommodative facility during school physical education classes. Overall, in ciliary-muscle exercises, the effects of the 1-s duration and the 3-s duration were better than that of the 5-s duration, which significantly reduced the growth rate of eye axial length and better improved the children's mild and moderate myopia. If children can actively apply this ciliary-muscle exercise along with physical activities to their daily life, they can improve their personal health and develop health-promoting behaviors, which would benefit them for life.

## Data availability statement

The raw data supporting the conclusions of this article will be made available by the authors, without undue reservation.

## Ethics statement

The studies involving human participants were reviewed and approved by the Ethics Committee of Soochow University (No. SUA20201010H01). Written informed consent to participate in this study was provided by the participants' legal guardian/next of kin.

## Author contributions

Conceptualization, validation, formal analysis, data curation, and writing—original draft preparation: SZ and MZ. Methodology: SZ. Investigation: WZ. Resources and project administration: RY. Writing—review and editing, supervision, and funding acquisition: RY and GC. Visualization: MZ. All authors have read and agreed to the published version of the manuscript.

## References

[1] World Health Organization. World Report on Vision. Geneva: World Health Organization. (2019).

[2] Hansen MH Laigaard PP Olsen EM Skovgaard AM Larsen M Kessel L. Low physical activity and higher use of screen devices are associated with myopia at the age of 16-17 years in the CCC2000 Eye Study. Acta Ophthalmol. (2020) 98:315–21. 10.1111/aos.14242 31502414

[3] Read SA Collins MJ. The short-term influence of exercise on axial length and intraocular pressure. Eye (Lond). (2011) 25:767–74. 10.1038/eye.2011.54 21423141 PMC3178122

[4] Jin G Pan JL Cai G. The practical significance and empirical research of physical exercise on the vision health of primary school students. J Cap Inst Phys Educ. (2021) 33:40–8.

[5] King K Chen J Chen G Tan Q Zhou S Pan J. Effect of three types of ball playing on kinetic visual acuity for children in junior grades. Chin J Rehab Theory Pract. (2019) 25:1279–82.

[6] Yin RB Guo M Zhang M Xu JR. Comparison of the degree of improvement of the visual health of sixth grade students by rubber board and sand board table tennis. Sport Res. (2022) 43:44–50. 10.12064/ssr.20220307

[7] Tsotsos JK Abid O Kotseruba I Solbach MD. On the control of attentional processes in vision. Cortex. (2021) 137:305–29. 10.1016/j.cortex.2021.01.001 33677138

[8] Radhakrishnan H Allen PM Charman WN. Dynamics of accommodative facility in myopes. Invest Ophthalmol Vis Sci. (2007) 48:4375–82. 10.1167/iovs.07-0269 17724230

[9] He ZY Ma SS Liang N. Principles of eye chart design. China Opt Technol Mag. (2004) 2004:77–80.

[10] Han L Li XR Wu SY. Development and application of a new near-use logarithmic vision chart. J Tianjin Med Univ. (2010) 16:134–7.36961662

[11] Yin R Xu J Wang H Zhou S Zhang M Cai G. Effect of physical activity combined with extra ciliary-muscle training on visual acuity of children aged 10-11. Front Public Health. (2022) 10:949130. 10.3389/fpubh.2022.949130 36111187 PMC9468474

[12] Wagner S Schaeffel F Zrenner E Straßer T. Prolonged nearwork affects the ciliary muscle morphology. Exp Eye Res. (2019) 186:107741. 10.1016/j.exer.2019.107741 31336108

[13] Chen JC Schmid KL Brown B. The autonomic control of accommodation and implications for human myopia development: a review. Ophthal Physiol Opt. (2003) 23:401–22. 10.1046/j.1475-1313.2003.00135.x 12950887

[14] Zhang M Fu Q Zhou S Tan Q Cai G. Meta-analysis on prevention and control of myopia in children and adolescents by physical activity. Sport Res. (2022) 43:55–64. 10.12064/ssr.2022010722809757

[15] Sun L Cai G Yin RB Pan JL Wang GX Chen G. Correlation between static visual acuity and dynamic visual acuity in children and its significance for physical activity. Chin Rehab Theory Pract. (2018) 24:1485–8. 10.3969/j.issn.1006-9771.2018.12.02519342241

[16] Oliveira C Tello C Liebmann JM Ritch R. Ciliary body thickness increases with increasing axial myopia. Am J Ophthalmol. (2005) 140:324–5. 10.1016/j.ajo.2005.01.047 16086961

[17] Yin RB Sun L Wang GX Cai G Huang K Yang T. Applying ICF theory to study the effect of physical activity on adolescent myopia. Chin Rehab Theory and Pract. (2018) 24:1223–7.

[18] Lu ZS Zhao XH Tan L. Vision training: methods and applications to prevent and control low vision in children and adolescents. J Shanghai Inst Phys Educ. (2020) 44:27–32. 10.16099/j.sus.2020.08.004

[19] Drexler W Findl O Schmetterer L Hitzenberger CK Fercher AF. Eye elongation during accommodation in humans: differences between emmetropes and myopes. Investigative Ophthalmol Vis Sci. (1998) 39:2140–7. 9761293

[20] Jeon S Lee WK Lee K Moon NJ. Diminished ciliary muscle movement on accommodation in myopia. Exp Eye Res. (2012) 105:9–14. 10.1016/j.exer.2012.08.014 23068564

[21] Hughes RPJ Read SA Collins MJ Vincent SJ. Axial elongation during short-term accommodation in myopic and nonmyopic children. Invest Ophthalmol Vis Sci. (2022) 63:12. 10.1167/iovs.63.3.12 35275173 PMC8934556

[22] Wylegała A. The effects of physical exercises on ocular physiology: a review. J Glaucoma. (2016) 25:e843–9. 10.1097/IJG.0000000000000454 27275655

[23] May PJ Reiner A Gamlin PD. Autonomic regulation of the eye. In: Oxford Research Encyclopedia of Neuroscience. Shapley R, editor. Oxford: Oxford University Press. (2019). 10.1093/acrefore/9780190264086.013.276

[24] Sander BP. The Influence of the Autonomic Nervous System on the Human Choroid [MSc thesis]. Brisbane, Australia: Queensland University of Technology. (2017).

